# Why choose solitude? Motivations of people with impaired personality functioning

**DOI:** 10.1192/j.eurpsy.2026.11005

**Published:** 2026-07-31

**Authors:** R. Heissler, N. Doubková, V. Juríčková, K. Knížková, M. Preiss, P. Mohr

**Affiliations:** 1 National Institute of Mental Health, Klecany, Czech Republic; 2 Center for Advanced Studies of Brain and Consciousness, National Institute of Mental Health, Klecany; 3 AMBIS University; 4 Department of Psychiatry, First Faculty of Medicine, Charles University and General University Hospital in Prague; 5 University of New York in Prague, Prague, Czech Republic

## Abstract

**Introduction:**

Solitude, understood as the pleasant experience of being alone, can represent a positive and meaningful aspect of life. It may be a source of autonomy, creativity, or personal growth. However, the motivations underlying solitude differ among individuals and may be shaped by personality functioning (PF). While some are drawn to solitude for self-determined reasons like self-reflection or growth, others may be motivated by non-self-determined motives such as anxiety or withdrawal tendencies, particularly relevant in the context of impaired PF.

**Objectives:**

The study aimed to examine motivational patterns for solitude in individuals with varying levels of PF, with a focus on those experiencing impaired functioning.

**Methods:**

A total of 1736 participants (1248 women, mean age 37.42 ± 12.14; 152 with severe/extreme PF impairment), divided into five levels of PF (from none to extreme), completed the Motivation for Solitude Scale–Short Form. Analyses included ANOVA to examine group differences and K-means clustering to identify motivational profiles related to solitude.

**Results:**

Differences in motivations have emerged, where individuals with more impaired PF were more likely to endorse anxiety-driven motivations for spending time alone. Additionally, four motivational clusters with two interconnected main dimensions have been identified: 1) solitude motivated by anxiety and discomfort when around others, and 2) solitude driven by the pursuit of self-reflection, or intrapersonal contact. Subgroups have been identified, and among those driven by anxiety, a subgroup also valued self-reflection and connection, while another did not. Similarly, among individuals without anxiety toward others, one subgroup emphasized self-reflection and connection over the other.

**Conclusions:**

The findings demonstrate that the solitude motivations of people with and without impaired PF are not uniform but are driven by the presence or absence of anxiety, and by valuing self-reflection and connection. For those with impaired personality functioning, anxiety-driven solitude was more common, but even then, half of them were able to use and value the solitude for self-reflection, creativity, and connection. The findings suggest a potential for promoting self-reflection and other self-determined drivers while being alone among those who do not yet value it, which could support their mental health and personal development.

**Acknowledgements:**

This study has been supported by the Czech Science Foundation under grant nr. 24-10889S.

**Disclosure of Interest:**

None Declared

